# Outcomes of an extended Morrow procedure without a concomitant mitral valve procedure for hypertrophic obstructive cardiomyopathy

**DOI:** 10.1038/srep29031

**Published:** 2016-06-30

**Authors:** Yun Liu, Yunhu Song, Ge Gao, Jun Ran, Wenjun Su, Haojie Li, Yajie Tang, Fujian Duan, Hansong Sun

**Affiliations:** 1 Department of Cardiovascular Surgery, Fuwai Hospital, National Center for Cardiovascular Diseases, Chinese Academy of Medical Sciences and Peking Union Medical College, Beijing, China

## Abstract

The indications for a concomitant mitral valve (MV) procedure remain controversial for patients with hypertrophic obstructive cardiomyopathy (HOCM). According to previous studies, a concomitant MV surgery was required in 11–20% of inpatient operations. Thus, we aimed to study the outcomes of an extended Morrow procedure without a concomitant MV procedure for HOCM patients who had no intrinsic abnormalities of the MV apparatus. We retrospectively reviewed 232 consecutive HOCM patients who underwent extended Morrow procedures from January 2010 to October 2014. Only 10 (4.31%) patients with intrinsic MV diseases underwent concomitant MV procedures. Of the 232 patients, 230 had no to mild mitral regurgitation (MR) postoperatively. We separated the 232 patients into two groups according to preoperative MR degree. One group is mild MR, and the other is moderate or severe MR. The three-month, one-year, and three-year composite end-point event-free survival rates had no difference between two groups (p = 0.820). When we separated the patients to postoperative no or trace MR group and mild MR group, there was also no difference on survival rates (p = 0.830). In conclusion, concomitant mitral valve procedures are not necessary for HOCM patients with MR caused by systolic anterior motion, even moderate to severe extent.

Hypertrophic cardiomyopathy has been recognized as the most common heritable cardiovascular disease, which affects one in 500 individuals^1^,^2^. Obstructive hypertrophic cardiomyopathy (HOCM) is one form of hypertrophic cardiomyopathy accompanied by the obstruction of the left ventricular outflow tract (LVOT). LVOT obstruction is an independent predictor of adverse outcomes^3^. A surgical septal myectomy is currently considered the gold standard treatment for HOCM patients with severe symptoms unresponsive to maximum-tolerated drug therapy^4^. Surgical procedures can successfully relieve a LVOT pressure gradient and associated mitral regurgitation (MR), which provide satisfactory long-term prognosis^5^.

However, the relative scarcity of experienced surgeons capable of performing a septal myectomy in developing countries impedes the expansion of this operation^6^. From January 2010 to October 2014, the surgical team of the ninth department of surgery in Fuwai hospital completed 232 consecutive extended Morrow procedures. Concomitant mitral valve (MV) procedures were not performed when there was no intrinsic MV pathological change or MR was caused by systolic anterior motion (SAM). Only 10 (4.31%) patients underwent a concomitant MV procedure. According to previous studies, a concomitant MV surgery was required in 11–20% of inpatient operations. However, evidence for the indication of MV surgery is scarce in these patients. Therefore, the purpose of the present study was to evaluate the mid-term effects of an extended Morrow procedure and compare the prognosis of patients with different MR stages when a concomitant MV procedure was only conducted in patients with pathological intrinsic MV changes. We also tried to identify the predictors of adverse prognosis.

## Results

### Baseline characteristics

The baseline characteristics were summarized in Table 1. The study cohort was predominantly male (57.8%) with an average age of 47.4 ± 12.2 years. The cohort included 57 (24.6%) patients with hypertension that was not clinically severe enough to cause the severe degree of left ventricular asymmetric hypertrophy (see Supplementary Table S2). Two hundred and seven (89.2%) of the patients in this study were in the New York Heart Association (NYHA) functional class III–IV before the operation. Preoperative atrial fibrillation (AF) documented by an electrocardiogram was found in 46 (19.8%) patients. Fifteen (6.5%) septal myectomy procedures were performed after an alcohol septal ablation for a significant residual LVOT pressure gradient under basal conditions or after exercise and heart failure symptoms or syncope refractory to pharmacologic therapy.

The preoperative echocardiographic data were also shown in Table 1. The maximum septum thickness was 25.5 ± 6.5 mm, the LVOT pressure gradient was 92.2 ± 26.4 mmHg, and the left atrial dimension was 41.7 ± 7.2 mm. SAM and MR were observed in all of the patients.

**Figure 1:**
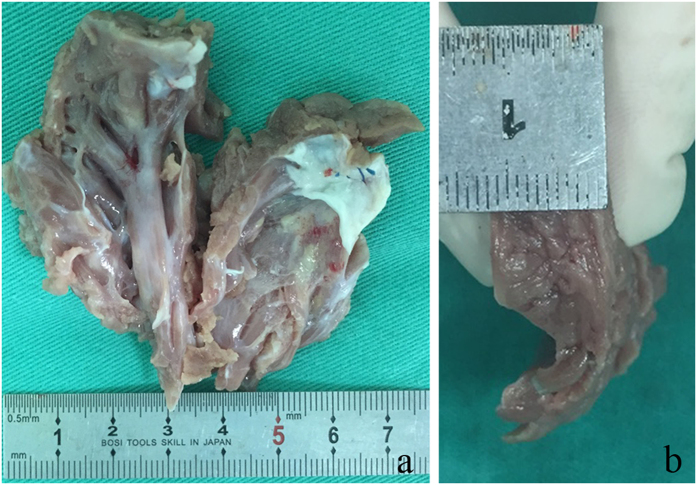
Excised cardiac myectomy specimens. To sufficiently expose and assess the excised thickness, Dr. Song divided the whole specimen in the middle during the surgery. (**a**) Antero-posterior view (weight 19.9 g), (**b**) lateral view of another specimen.

The number of operations distributed unequally during five years and increased tremendously. Concomitant procedures were conducted in 69 (29.7%) patients, including coronary artery bypass grafting (CABG) (54), MV repair (3), MV replacement (6), CABG and MV replacement both (1), the reduction of right ventricular outflow obstruction (2), and others (3). An adjunctive surgical ablation of AF was conducted in only five patients with persistent AF. Table 2 shows the number and reasons of the concomitant MV procedures. The mean weight of the excised myocardium of the last 90 patients who had surgery was 9.28 ± 3.38 g (Fig. 1).

### Perioperative results

There were no deaths within 30 days after the operation (operative mortality, 0%). The interventricular septum thickness was significantly reduced from 25.5 ± 6.5 mm before surgery to 17.1 ± 4.8 mm (P < 0.001) pre-discharge. Simultaneously, the LVOT pressure gradient fell from 92.2 ± 26.4 mmHg to 15.2 ± 8.7 mmHg (P < 0.001).

The postmyectomy transesophageal echocardiography intraoperatively identified iatrogenic ventricular septal defects (VSDs) that were immediately repaired in three (1.3%) patients who were discharged uneventfully. A perforation of the anterolateral free wall near the apex contiguous to the anterior septum was created in one patient. It was located promptly by the surgeon and was repaired without sequela. Neither iatrogenic injury of the MV nor iatrogenic injury of the aortic valve occurred in the 232 procedures. The mean cardiopulmonary bypass and aortic cross-clamp times were 83.4 ± 28.7 and 53.9 ± 19.0 minutes. The median, 25th, 75th percentiles for the postoperative intubation time were 15 hours, 12 hours, and 18 hours, respectively.

Six (2.6%) patients received new permanent pacemakers before being discharged from the hospital because of a CAVB. Of these six patients, two had a preoperative complete right bundle branch block (CRBBB), one underwent a concomitant reduction of a right ventricular outflow obstruction, and two had an iatrogenic VSD during the operation. Almost all of the patients had no to mild MR postoperatively, except for two patients who maintained moderate MR. However, among the 101 patients with mild MR preoperatively, 51 (50.5%) patients had no or trace MR, and 49 (48.5%) had mild MR. In contrast, among the 131 patients with moderate to severe MR preoperatively, only 39 (29.8%) had no or trace MR, and 91 (69.5%) had mild MR. The patients with mild MR preoperatively had less MR postoperatively (p = 0.002).

### Follow-up results

The mean clinical follow-up period was 23.8 ± 13.7 months (one to 59 months). A total of 228 (98.3%) patients accepted our telephone questionnaire. This group of patients experienced substantial symptomatic and hemodynamic improvement after the myectomy. During the follow-up, 193 (83.2%) patients remained asymptomatic or minimally symptomatic. The degree of LVOT pressure gradients decreased significant compared with the preoperative values (12.9 ± 10.1 mmHg at a recent evaluation, P < 0.001). The left atrial dimension fell to 38.7 ± 5.9 mm (P < 0.001). Eleven (4.7%) patients had a residual maximal LVOT pressure gradient >30 mmHg, and two (0.8%) patients had a residual maximal LVOT pressure gradient >50 mmHg. These appeared to be related to a residual obstruction of an initial myectomy rather than recurrent obstruction from muscle regrowth. All of the patients are free from any cardiac reoperation to date. Of the 46 patients with preoperative AF documented by an electrocardiogram, only 26 (56.5%) had a normal sinus rhythm during the follow-up.

**Figure 2:**
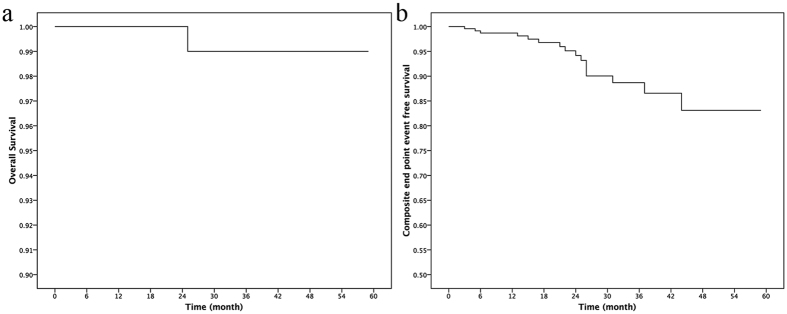
(**a**) Survival Kaplan-Meier curve of the 232 patients, (**b**) composite end-point event-free survival curve.

To date, only one patient has died. The overall survival was 100% at three months, 100% at one year, and 99.0% at three years (Fig. 2a). The one death was a sudden cardiac death occurring 25 months after surgery. This patient was a 28-year-old woman with a global extreme hypertrophied left ventricular and a 46-mm-thick interventricular septum.

During the follow-up, 16 (6.9%) patients met the composite end-point (Table 3). The three-month, one-year, and three-year end-point event-free survival rates were 99.6%, 98.7%, and 88.7%, respectively (Fig. 2b). The most common reason for rehospitalization was heart failure symptoms (12, 75.0%). Of the 12 patients, seven were rehospitalized only once due to pneumonia and secondary reversible acute heart failure syndrome. Two patients had a residual LVOT pressure gradient >50 mmHg. The other three patients had chronic heart failure and presented with episodes of decompensation. Of these three patients with chronic heart failure, one had iatrogenic VSD and CAVB in operation. One patient had coronary artery disease, and heart failure was always secondary to paroxysmal AF. The last patient had a complete left bundle branch block in operation, and there was a gradual cardiac enlargement after discharge. Table 3 depicts all of the end-point events.

### Comparison between the patients with preoperative mild MR and moderate to severe MR

**Figure 3:**
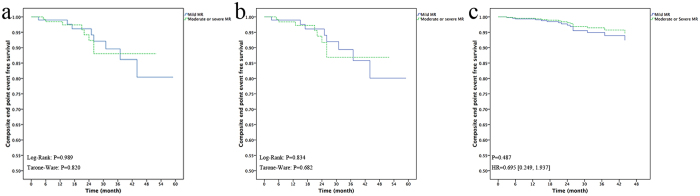
Survival-free from composite end-point events in the two subgroups: mild mitral regurgitation (MR) preoperatively, moderate or severe MR preoperatively. (**a**) Kaplan-Meier curve of all of the patients, (**b**) Kaplan-Meier curve of 222 patients, excluding 10 with a mitral valve procedure, (**c**) survival adjusted for left atrial diameter, left ventricular outflow tract pressure gradient, and percutaneous transluminal septal myocardial ablation history.

We separated the 232 patients into two groups according to the MR stage before the operation. One group included 17 patients with severe MR and 114 patients with moderate MR. The other group included 101 patients with mild MR. There were no differences in the baseline clinical characteristics between the two groups, except for the left atrial diameter, LVOT pressure gradient, and percutaneous transluminal septal myocardial ablation (PTSMA) history (Table 1). The three-month, one-year, and three-year composite end-point event-free survival rates were 100%, 98.5%, 88.0% and 99.0%, 99.0%, 89.6%, respectively for the two groups (Tarone-Ware p = 0.820) (Fig. 3a). When we excluded the 10 patients who underwent the concomitant MV procedure, there was also no difference (100%, 98.4%, 86.9% and 99.0%, 99.0%, 89.4%, respectively, for the two groups, Tarone-Ware p = 0.682) (Fig. 3b). If we adjusted the composite end-point event-free survival rates for the left atrial diameter, the LVOT pressure gradient, and the PTSMA history, then the differences between the two groups were not statistically significant (p = 0.487) (Fig. 3c).

### Comparison between the patients with no or trace MR and mild MR postoperatively

**Figure 4:**
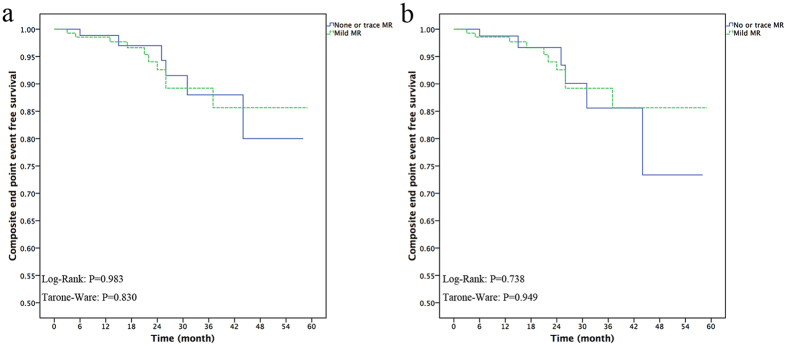
Survival-free from the composite end-point events in the two subgroups: no or trace mitral regurgitation (MR) postoperatively, mild MR postoperatively. (**a**) Kaplan-Meier curve of all of the patients, (**b**) Kaplan-Meier curve of 222 patients, excluding 10 with mitral a valve procedure.

In this study, the patients with moderate to severe MR preoperatively were more likely to have residual mild MR postoperatively. To identify the influence of postoperative mild MR on the outcomes, we excluded the two patients who had moderate MR postoperatively. We separated the 230 patients into two groups: 90 (39.1%) patients with no MR or trace MR and 140 (60.9%) patients with mild MR. The three-month, one-year, and three-year composite end-point event-free survival rates of the patients with mild MR were similar to those of the patients with no or trace MR (100%, 98.9%, 88.0% vs. 99.3%, 98.6%, 89.2%, respectively; Tarone-Ware p = 0.830) (Fig. 4a). When we excluded the 10 patients who underwent the concomitant MV procedure, there was also no difference (Tarone-Ware p = 0.949) (Fig. 4b).

### Predictors of end-point events

In the Cox proportional hazard models for the composite end-points, we included all 232 patients in the analysis. The data from the univariable Cox analysis are described in Table 4. Sex and variables with P < 0.05 were then entered into a stepwise multivariable analysis (Table 4). Old age, iatrogenic VSD, and postoperative AF were identified as independent predictors of worse outcomes in HOCM patients undergoing surgical treatment.

## Discussion

Dr. W. P. Cleland, a British surgeon, reported the first myotomy in 1958^7^. Thereafter, the surgical treatment for HOCM was developed at several North American and European centers^8^,^9^,^10^. Currently, the most widely used surgical method is myectomy via an aortic approach, which is called an extended Morrow procedure^11^. Recently, the European Society of Cardiology (ESC) guideline and American College of Cardiology Foundation (ACC)/American Heart Association (AHA) guidelines on the management of HCM stated that a surgical septal myectomy is the gold standard and primary treatment option for patients with HOCM and severe symptoms of heart failure^12^,^13^.

The outcomes of the septal myectomy in our consecutive 232 HOCM patients were as good as those reported by other famous centers^5^ with no perioperative death, while one HCM-related death occurred during the follow-up, and a total of 16 (6.9%) HCM-related rehospitalizations occurred after discharge. One hundred ninety-three (83.2%) of the patients remained asymptomatic or minimally symptomatic (NYHA class I) during the intermediate follow-up.

Although a transaortic septal myectomy has a good prognosis for HOCM patients^5^,^10^, some controversy remains concerning the surgery, especially the management of MR^14^. Dr. Klues recognized that there were diverse structural MV alterations in HOCM^15^. Several adjunctive procedures, including realignment of papillary muscles^16^, anterior mitral leaflet plication^17^, and anterior mitral leaflet extension^18^, are used to eliminate SAM and decrease MR. Our study suggests that a concomitant MV procedure is not necessary for HOCM patients without intrinsic MV disease. On one hand, an extended Morrow procedure alone is effective to alleviate even moderate to severe MR caused by SAM. Only 10 (4.3%) patients of this population underwent a concomitant MV procedure, which is less than that previous reported^19^. In 230 (99.1%) of 232 patients, the MR was decreased to mild or less postoperatively. Of the 131 patients with moderate or severe MR preoperatively, 130 (99.2%) had no to mild MR postoperatively. On the other hand, mild MR postoperatively was acceptable and did not increase the adverse cardiovascular event rate. During the follow-up, there was no difference in end-point events-free survival between the patients with no or trace MR and patients with mild MR. Thus, the sufficient elimination of LVOT obstruction and an appropriate dissociation of abnormal attachments provided excellent surgery outcomes for HOCM patients with no intrinsic pathological MV changes. A resection distally toward the left ventricular apex as far as possible is important. A normal left ventricle geometry created by surgery may improve the flow of blood during the period from end diastole to isovolumic systole to eliminate SAM^20^. Dissociation at the base of the papillary muscles from around hypertrophy myocardial tissue and the excision of abnormal fibrous or muscular attachment structures can result in fair mobility of the papillary muscle and the anterior leaflet of MV^14^.

Both preoperative AF and postoperative AF are predictors of composite end-point events. Postoperative AF is an independent predictor of the composite end-point events. In other studies, AF was always identified as the risk factor of worse outcomes^21^; however, the reason is not entirely clear. The residual AF, which induces a loss of atrial mechanical function, can indeed reduce cardiac output^22^. Some reports demonstrate that AF could trigger ventricular tachycardia^23^. In this cohort, adjunctive surgical ablation of AF was conducted in only five patients with persistent AF. All five patients maintained sinus rhythm during the follow-up with no end point events. Therefore, a MAZE procedure might be conducted in every patient with AF ever recorded by electrocardiogram. Old age was always determined as a risk factor associated with worse outcomes^24^. However, contrary to other studies, we found that procedures performed concomitant with the myectomy did not increase the possibility of the composite end-point events^25^.

There are still some limitations. This is a retrospective study; all of the surgeries were performed by one experienced surgical team. There was no concomitant MV procedure, such as a leaflet plication or anterior leaflet extension, conducted to eliminate SAM and MR. Nevertheless, these conclusions still may be valid and clinically relevant. As previously reported, the survival rate of the HOCM patients after surgery was excellent. However, more attention should be focus on HOCM-related rehospitalization. The question of whether surgery could abolish the risk for progression to the end-stage phase of heart failure requires further study.

Our results prove that the extended Morrow procedure can successfully improve the symptoms of HOCM patients. Concomitant MV procedures are not necessary for patients who have even moderate to severe MR caused by SAM. Residual mild MR postoperatively does not influence the outcome of these patients. Old age, iatrogenic SVD, and postoperative AF are independent predictors of worse outcomes.

## Methods

### Study population

This study was approved by the Human Research Ethics Committee of the Fuwai Hospital and was performed in accordance with the Declaration of Helsinki and the approved guidelines. Oral informed consent was obtained from all of the patients via a telephone questionnaire. We retrospectively reviewed all of the 232 consecutive HOCM patients who underwent a transaortic extended septal myectomy in the ninth department of surgery in our institution from January 2010 to October 2014. The diagnosis of HCM was based on clinical and echocardiographic features with the presence of a hypertrophied non-dilated left ventricular in the absence of other cardiac or systemic disease capable of producing the magnitude of the hypertrophy evident^26^. These patients had severe symptoms and an LVOT pressure gradient greater than 50 mmHg at rest or after exercise, despite treatment with a β-blocker, diltiazem, singly or in combination titrated to a maximum tolerated dose. Intrinsic MV pathological changes were defined as rheumatic disease, degeneration, prolapse, and infective endocarditis. This study was approved by the Fuwai Institutional Review Board.

### Echocardiographic evaluation

All of the patients underwent comprehensive echocardiogram examinations with commercially available instruments. All of the transthoracic echocardiography procedures were performed by the same professional doctor. Echocardiographic parameters were acquired using standard views and protocols^27^. The LV wall thickness was measured at the end-diastole. The LVOT peak velocity was measured with a continuous-wave Doppler, and the LVOT pressure gradient was estimated using a simplified Bernoulli equation^28^. The exercise-induced LVOT pressure gradient was assessed in patients with resting LVOT pressure gradients <50 mmHg. MR was graded by a color flow Doppler method according to the regurgitant jet area^29^ (see Supplementary Table S1).

### Transaortic septal myectomy

An intraoperative transesophageal echocardiography is a pre-requisite for surgery because it allows the detection of the ventricular septal anatomy and its thickness, the evaluation of the MV function, and the identification of other abnormalities. The chest is opened with a complete standard median sternotomy. To sufficiently expose the subaortic septum, an oblique aortotomy much closer to the sinotubular junction than that usually performed for an aortic valve replacement is made and carried to the midpoint of the noncoronary sinus^30^. We begin the first incision from the basal region toward the apex of the heart approximately 5 mm left of the membranous septum to avoid complete atrioventricular block (CAVB). The incision is extended leftward to the anterolateral free wall of the left ventricle near the MV annulus. The trough is then continued in the apical direction beyond the bases of the papillary muscles. Importantly, all of the papillary muscle fusions to the septum or ventricular free wall are divided to increase mobility of the papillary muscle. Abnormal chordal structure and anomalous fibrous or muscle attachments of the MV apparatus to the ventricular septum should be excised. Before the patient is weaned from cardiopulmonary bypass, a transesophageal echocardiography is repeated to remeasure the LVOT pressure gradient, the continuity of the septum and the MV function.

### Follow-up

All of the follow-up data were obtained between January and May 2015. During the follow-up, the time and cause of death were provided to the immediate families. The end-point was a composite of all causes of death, resuscitation from sudden death, syncope, documented stroke, arrhythmia or congestive heart failure requiring hospitalization^25^. HCM-related death was defined as the sudden death, death or heart transplantation as a result of progressive heart failure. The definition of sudden death was an unexpected sudden collapse occurring <1 hour from the onset of symptoms in patients who had previously experienced a relatively stable or uneventful clinical course^25^.

### Statistical Analysis

Continuous data were expressed as the mean ± standard deviation and compared using a Student’s *t* test or analysis of variance. Categorical data were expressed as percentages and were compared with a chi-square test. A Cox proportional hazards model was used to identify the risk factors of composite end-points. A stepwise multivariable Cox proportional hazards model was developed to determine the independent risk factors. The proportional hazards assumption in the Cox model was assessed with models including time-by-covariate interactions. Cumulative event rates were calculated using a Kaplan-Meier method, and different event curves of outcomes were compared using a Log-Rank or Tarone-Ware test. All of the P values were reported two-sided, and P < 0.05 was considered statistically significant. Statistical analyses were performed using SPSS version 20 (IBM SPSS Inc., Chicago, IL).

## Additional Information

**How to cite this article**: Liu, Y. *et al.* Outcomes of an extended Morrow procedure without a concomitant mitral valve procedure for hypertrophic obstructive cardiomyopathy. *Sci. Rep.* **6**, 29031; doi: 10.1038/srep29031 (2016).

## Supplementary Material

Supplementary Information

## Figures and Tables

**Table 1:** Preoperative Baseline Clinical and Demographic Characteristics of Patients.

Parameters	All patients	Preoperative mild MR	Preoperative moderate or severe MR	P Value
Age (yrs)	47.4 ± 12.2	45.9 ± 12.2	48.5 ± 12.2	0.112
Males	57.8 (134/232)	64.4 (65/101)	52.7 (69/131)	0.082
NYHA functional class				0.931
I	0 (0/232)	0 (0/101)	0 (0/131)	
II	10.8 (25/232)	9.90 (10/101)	11.5 (15/131)	
III	82.8 (192/232)	83.2 (84/101)	82.4 (108/131)	
IV	6.46 (15/232)	6.93 (7/101)	6.11 (8/131)	
Atrial fibrillation	19.8 (46/232)	18.8 (19/101)	20.6 (27/131)	0.744
Symptom				
Dyspnea	90.5 (210/232)	87.1 (88/101)	93.1 (122/131)	0.174
Angina	38.4 (89/232)	44.6 (45/101)	33.6 (44/131)	0.103
Palpitation	40.5 (94/232)	38.6 (39/101)	42.0 (55/131)	0.686
Dizziness	45.3 (105/232)	50.5 (51/101)	41.2 (54/131)	0.184
Syncope	29.3 (68/232)	31.7 (32/101)	27.5 (36/131)	0.561
Echocardiography				
Max septum thickness (mm)	25.5 ± 6.49	25.5 ± 6.68	25.4 ± 6.37	0.968
Left atrial diameter (mm)	41.7 ± 7.19	40.4 ± 6.69	42.7 ± 7.44	0.018
LVEDD (mm)	41.2 ± 5.06	40.7 ± 4.81	41.5 ± 5.22	0.232
LVEF (%)	73.5 ± 6.90	73.8 ± 7.97	73.2 ± 5.97	0.559
LVOTPG (mmHg)	92.2 ± 26.4	87.1 ± 27.1	96.1 ± 25.4	0.009
Heart rate (beats/min)	65.4 ± 5.50	66.2 ± 5.90	64.8 ± 5.12	0.054
Systolic blood pressure (mmHg)	111 ± 12.4	112 ± 11.7	110 ± 12.9	0.253
Diastolic blood pressure (mmHg)	68.8 ± 8.99	69.2 ± 9.07	68.5 ± 8.96	0.565
Total cholesterol (mmol/L)	4.52 ± 0.99	4.45 ± 0.95	4.58 ± 1.01	0.314
Triglycerides (mmol/L)	1.62 ± 1.18	1.70 ± 1.42	1.57 ± 0.96	0.412
LDL cholesterol (mmol/L)	2.76 ± 0.84	2.70 ± 0.86	2.81 ± 0.82	0.351
HDL cholesterol (mmol/L)	1.16 ± 0.35	1.13 ± 0.31	1.19 ± 0.37	0.219
Fasting plasma glucose (mmol/L)	5.08 ± 0.82	5.07 ± 0.93	5.09 ± 0.73	0.857
Serum creatinine (μmol/L)	73.4 ± 18.5	73.5 ± 20.9	73.4 ± 16.4	0.972
Hypertension	24.6 (57/232)	18.8 (19/101)	29.0 (38/131)	0.091
Diabetes	5.60 (13/232)	5.94 (6/101)	5.34 (7/131)	1.000
Hemoglobin A1c (%)^a^	7.47 ± 1.29	7.38 ± 1.30	7.60 ± 1.47	0.812
Fasting plasma glucose (mmol/L)^a^	6.85 ± 1.58	6.82 ± 2.35	6.88 ± 0.62	0.948
CRBBB	3.88 (9/232)	3.96 (4/101)	3.82 (5/131)	1.000
I atrioventricular block	5.17 (12/232)	5.94 (6/101)	4.58 (6/131)	0.767
PTSMA history	6.46 (15/232)	10.9 (11/101)	3.05 (4/131)	0.028
Use of medication before admission				
Anti-HCM drugs	100 (232/232)	100 (101/101)	100 (131/131)	
β-blocker	99.1 (230/232)	100 (101/101)	98.5 (129/132)	0.506
Diltiazem	32.3 (75/232)	31.7 (32/101)	32.8 (43/131)	0.888
Diuretics^b^	7.8 (18/232)	6.9 (7/101)	8.4 (11/131)	0.806
Antihypertensive drugs				
ACE inhibitors	8.2 (19/232)	9.9 (10/101)	6.9 (9/131)	0.472
Dihydropyridine derivatives	8.6 (20/232)	10.9 (11/101)	6.9 (9/131)	0.347
Diuretics^c^	5.6 (13/232)	3.0 (3/101)	7.6 (10/131)	0.156
Anti-diabetic drugs				
Insulin	1.3 (3/232)	2.0 (2/101)	0.8 (1/131)	0.581
Oral anti-diabetic drugs	5.2 (12/232)	5.9 (6/101)	4.6 (6/131)	0.767
Isolated myectomy	70.3 (163/232)	67.3 (68/101)	72.5 (95/131)	0.469
Concomitant mitral valve procedure				0.302
None	95.7 (222/232)	98.0 (99/101)	93.9 (123/131)	
Mitral valvuloplasty	1.29 (3/232)	0 (0/101)	2.29 (3/131)	
Mitral valve replacement	3.02 (7/232)	1.98 (2/101)	3.82 (5/131)	

ACE, angiotensin-converting enzyme; CRBBB, complete right bundle branch block; HDL, high density lipoprotein; LDL, low density lipoprotein; LVEDD, left ventricular end diastolic diameter; LVEF, left ventricular ejection fraction; LVOTPG, left ventricular outflow tract pressure gradient; MR, mitral regurgitation; NYHA, New York Heart Association; PTSMA, percutaneous transluminal septal myocardial ablation.

Values are shown as mean ± SD or % (n/N).

^a^Values for patients with diabetes only.

^b^Diuretics for patients with heart failure and water sodium retention.

^c^Diuretics for patients with hypertension.

**Table 2:** Concomitant Mitral Valve Procedures and Reasons for the Procedure.

	Prolapse	Degeneration	RHD	SBE	ALL
MVR	0 (0/232)	0.4 (1/232)	1.3 (3/232)	1.3 (3/232)	3.0 (7/232)
MVP	1.3 (3/232)	0 (0/232)	0 (0/232)	0 (0/232)	1.3 (3/232)
All	1.3 (3/232)	0.4 (1/232)	1.3 (3/232)	1.3 (3/232)	4.3 (10/232)

MVP, mitral valvuloplasty; MVR, mitral valve replacement; RHD, rheumatic heart disease; SBE, subacute bacterial endocarditis.

Values are shown as % (n/N).

**Table 3:** Patients who met the composite end-point events.

End-point event	All patients	Mild MR preoperative	Moderate or severe MR preoperative
Heart failure	5.2 (12/232)	5.0 (5/101)	5.3 (7/131)
Sudden cardiac death	0.4 (1/232)	1.0 (1/101)	0 (0/131)
Syncope	0.4 (1/232)	1.0 (1/101)	0 (0/131)
Stroke	0.4 (1/232)	1.0 (1/101)	0 (0/131)
Peripheral embolism	0.4 (1/232)	0 (0/101)	0.8 (1/131)
All	6.9 (16/232)	7.9 (8/101)	6.1 (8/131)

MR, mitral regurgitation.

Values are shown as % (n/N).

**Table 4:** Cox proportional hazard analysis for the composite end-point events.

Variable	Univariable		Stepwise Multivariable	
	HR (95% CI)	P Value	HR (95% CI)	P Value
Age	1.061 (1.008–1.117)	0.024	1.071 (1.008–1.139)	0.028
Sex	0.915 (0.332–2.521)	0.863		0.826
Dyspnea	1.278 (0.168–9.705)	0.813		
Angina	1.226 (0.456–3.293)	0.687		
Palpitation	0.876 (0.318–2.417)	0.798		
Dizziness	1.499 (0.543–4.141)	0.435		
Syncope	1.824 (0.659–5.049)	0.247		
Atrial fibrillation preoperative	4.383 (1.636–11.74)	0.003		0.286
NYHA class	1.298 (0.170–9.919)	0.801		
Heart rate	1.071 (0.985–1.164)	0.108		
Systolic blood pressure	1.004 (0.965–1.043)	0.857		
Diastolic blood pressure	0.992 (0.939–1.049)	0.783		
Total cholesterol	1.086 (0.638–1.848)	0.762		
Triglycerides	0.559 (0.242–1.291)	0.173		
LDL cholesterol	1.311 (0.725–2.371)	0.371		
HDL cholesterol	1.335 (0.266–6.702)	0.726		
Fasting plasma glucose	0.881 (0.435–1.783)	0.725		
Serum creatinine	1.007 (0.987–1.027)	0.501		
Hypertension	0.795 (0.226–2.794)	0.720		
Hyperlipoidemia	0.948 (0.306–2.942)	0.927		
Diabetes	0.046 (0.000–652.6)	0.527		
PTSMA history	0.045 (0.000–399.4)	0.504		
CRBBB	3.710 (0.474–29.02)	0.212		
I atrioventricular block	3.187 (0.716–14.20)	0.128		
Max septum thickness	1.005 (0.932–1.083)	0.897		
LVOTPG	1.004 (0.987–1.020)	0.660		
Left atrial diameter	1.067 (1.004–1.134)	0.038		0.231
LVEDD	1.114 (1.026–1.209)	0.010		0.098
LVEF	0.975 (0.920–1.034)	0.398		
MR preoperative	0.993 (0.371–2.662)	0.989		
Isolated myectomy	1.008 (0.324–3.135)	0.990		
Iatrogenic CAVB	8.979 (1.997–40.37)	0.004		0.183
Iatrogenic VSD	19.41 (4.281–87.98)	<0.001	20.47 (4.276–98.01)	<0.001
Iatrogenic CLBBB	1.208 (0.444–3.284)	0.712		
Atrial fibrillation postoperative	4.989 (1.840–13.53)	0.002	5.016 (1.828–13.76)	0.002
MR postoperative	1.007 (0.366–2.775)	0.989		

CAVB, complete atrioventricular block; CLBBB, complete left bundle branch block; CRBBB, complete right bundle branch block; HDL, high density lipoprotein; HR, hazard ratio; LDL, low density lipoprotein; LVEDD, left ventricular end diastolic diameter; LVEF, left ventricular ejection fraction; LVOTPG, left ventricular outflow tract pressure gradient; MR, mitral regurgitation; NYHA, New York Heart Association; PTSMA, percutaneous transluminal septal myocardial ablation; VSD, ventricular septal defect.

## References

[b1] Kramer C. M. *et al.* Hypertrophic Cardiomyopathy Registry: The rationale and design of an international, observational study of hypertrophic cardiomyopathy. American heart journal 170, 223–230, doi: 10.1016/j.ahj.2015.05.013 (2015). 26299218 PMC4548277

[b2] Maron B. J. *et al.* Contemporary definitions and classification of the cardiomyopathies: an American Heart Association Scientific Statement from the Council on Clinical Cardiology, Heart Failure and Transplantation Committee; Quality of Care and Outcomes Research and Functional Genomics and Translational Biology Interdisciplinary Working Groups; and Council on Epidemiology and Prevention. Circulation 113, 1807–1816, doi: 10.1161/circulationaha.106.174287 (2006). 16567565

[b3] Maron M. S. *et al.* Effect of left ventricular outflow tract obstruction on clinical outcome in hypertrophic cardiomyopathy. The New England journal of medicine 348, 295–303, doi: 10.1056/NEJMoa021332 (2003). 12540642

[b4] Brown M. L. & Schaff H. V. Surgical management of obstructive hypertrophic cardiomyopathy: the gold standard. Expert review of cardiovascular therapy 6, 715–722, doi: 10.1586/14779072.6.5.715 (2008). 18510487

[b5] Maron B. J. *et al.* Hypertrophic Cardiomyopathy in Adulthood Associated With Low Cardiovascular Mortality With Contemporary Management Strategies. Journal of the American College of Cardiology 65, 1915–1928, doi: 10.1016/j.jacc.2015.02.061 (2015). 25953744

[b6] Maron B. J., Rastegar H., Udelson J. E., Dearani J. A. & Maron M. S. Contemporary surgical management of hypertrophic cardiomyopathy, the need for more myectomy surgeons and disease-specific centers, and the Tufts initiative. The American journal of cardiology 112, 1512–1515, doi: 10.1016/j.amjcard.2013.06.040 (2013). 24012037

[b7] Goodwin J. F., Hollman A., Cleland W. P. & Teare D. Obstructive cardiomyopathy simulating aortic stenosis. British heart journal 22, 403–414 (1960). 13851098 10.1136/hrt.22.3.403 PMC1017672

[b8] Parry D. J. *et al.* Short and medium term outcomes of surgery for patients with hypertrophic obstructive cardiomyopathy. The Annals of thoracic surgery 99, 1213–1219, doi: 10.1016/j.athoracsur.2014.11.020 (2015). 25678500

[b9] Iacovoni A. *et al.* A contemporary European experience with surgical septal myectomy in hypertrophic cardiomyopathy. European heart journal 33, 2080–2087, doi: 10.1093/eurheartj/ehs064 (2012). 22522842 PMC3418509

[b10] Ommen S. R. *et al.* Long-term effects of surgical septal myectomy on survival in patients with obstructive hypertrophic cardiomyopathy. Journal of the American College of Cardiology 46, 470–476, doi: 10.1016/j.jacc.2005.02.090 (2005). 16053960

[b11] Maron B. J. *et al.* American College of Cardiology/European Society of Cardiology Clinical Expert Consensus Document on Hypertrophic Cardiomyopathy. A report of the American College of Cardiology Foundation Task Force on Clinical Expert Consensus Documents and the European Society of Cardiology Committee for Practice Guidelines. European heart journal 24, 1965–1991 (2003). 14585256 10.1016/s0195-668x(03)00479-2

[b12] Elliott P. M. *et al.* 2014 ESC Guidelines on diagnosis and management of hypertrophic cardiomyopathy: the Task Force for the Diagnosis and Management of Hypertrophic Cardiomyopathy of the European Society of Cardiology (ESC). European heart journal 35, 2733–2779, doi: 10.1093/eurheartj/ehu284 (2014). 25173338

[b13] Gersh B. J. *et al.* 2011 ACCF/AHA Guideline for the Diagnosis and Treatment of Hypertrophic Cardiomyopathy: a report of the American College of Cardiology Foundation/American Heart Association Task Force on Practice Guidelines. Developed in collaboration with the American Association for Thoracic Surgery, American Society of Echocardiography, American Society of Nuclear Cardiology, Heart Failure Society of America, Heart Rhythm Society, Society for Cardiovascular Angiography and Interventions, and Society of Thoracic Surgeons. Journal of the American College of Cardiology 58, e212–260, doi: 10.1016/j.jacc.2011.06.011 (2011). 22075469

[b14] Dearani J. A., Ommen S. R., Gersh B. J., Schaff H. V. & Danielson G. K. Surgery insight: Septal myectomy for obstructive hypertrophic cardiomyopathy–the Mayo Clinic experience. Nature clinical practice. Cardiovascular medicine 4, 503–512, doi: 10.1038/ncpcardio0965 (2007). 17712363

[b15] Klues H. G., Maron B. J., Dollar A. L. & Roberts W. C. Diversity of structural mitral valve alterations in hypertrophic cardiomyopathy. Circulation 85, 1651–1660 (1992). 1572023 10.1161/01.cir.85.5.1651

[b16] Teo E. P., Teoh J. G. & Hung J. Mitral valve and papillary muscle abnormalities in hypertrophic obstructive cardiomyopathy. Current opinion in cardiology 30, 475–482, doi: 10.1097/hco.0000000000000200 (2015). 26192489

[b17] Balaram S. K. *et al.* Role of mitral valve plication in the surgical management of hypertrophic cardiomyopathy. The Annals of thoracic surgery 94, 1990–1997; discussion 1997–1998, doi: 10.1016/j.athoracsur.2012.06.008 (2012). 22858269

[b18] Vriesendorp P. A. *et al.* Long-term benefit of myectomy and anterior mitral leaflet extension in obstructive hypertrophic cardiomyopathy. The American journal of cardiology 115, 670–675, doi: 10.1016/j.amjcard.2014.12.017 (2015). 25591899

[b19] Yu E. H. *et al.* Mitral regurgitation in hypertrophic obstructive cardiomyopathy: relationship to obstruction and relief with myectomy. Journal of the American College of Cardiology 36, 2219–2225 (2000). 11127464 10.1016/s0735-1097(00)01019-6

[b20] Ro R. *et al.* Vector flow mapping in obstructive hypertrophic cardiomyopathy to assess the relationship of early systolic left ventricular flow and the mitral valve. Journal of the American College of Cardiology 64, 1984–1995, doi: 10.1016/j.jacc.2014.04.090 (2014). 25440093

[b21] Siontis K. C. *et al.* Atrial fibrillation in hypertrophic cardiomyopathy: prevalence, clinical correlations, and mortality in a large high-risk population. Journal of the American Heart Association 3, e001002, doi: 10.1161/jaha.114.001002 (2014). 24965028 PMC4309084

[b22] Andrade J., Khairy P., Dobrev D. & Nattel S. The clinical profile and pathophysiology of atrial fibrillation: relationships among clinical features, epidemiology, and mechanisms. Circulation research 114, 1453–1468, doi: 10.1161/circresaha.114.303211 (2014). 24763464

[b23] Limongelli G. *et al.* Noninvasive risk stratification prevents sudden death due to paroxysmal atrial fibrillation in hypertrophic cardiomyopathy. Journal of cardiovascular medicine (Hagerstown, Md.) 7, 711–713, doi: 10.2459/01.JCM.0000243007.97793.0f (2006). 16932087

[b24] Panaich S. S. *et al.* Results of ventricular septal myectomy and hypertrophic cardiomyopathy (from Nationwide Inpatient Sample [1998-2010]). The American journal of cardiology 114, 1390–1395, doi: 10.1016/j.amjcard.2014.07.075 (2014). 25205630

[b25] Desai M. Y. *et al.* Predictors of long-term outcomes in symptomatic hypertrophic obstructive cardiomyopathy patients undergoing surgical relief of left ventricular outflow tract obstruction. Circulation 128, 209–216, doi: 10.1161/circulationaha.112.000849 (2013). 23770748

[b26] Maron B. J. & Maron M. S. Hypertrophic cardiomyopathy. Lancet 381, 242–255, doi: 10.1016/s0140-6736(12)60397-3 (2013). 22874472

[b27] Afonso L. C., Bernal J., Bax J. J. & Abraham T. P. Echocardiography in hypertrophic cardiomyopathy: the role of conventional and emerging technologies. JACC. Cardiovascular imaging 1, 787–800, doi: 10.1016/j.jcmg.2008.09.002 (2008). 19356516

[b28] Sasson Z., Yock P. G., Hatle L. K., Alderman E. L. & Popp R. L. Doppler echocardiographic determination of the pressure gradient in hypertrophic cardiomyopathy. Journal of the American College of Cardiology 11, 752–756 (1988). 3351141 10.1016/0735-1097(88)90207-0

[b29] Zoghbi W. A. *et al.* Recommendations for evaluation of the severity of native valvular regurgitation with two-dimensional and Doppler echocardiography. Journal of the American Society of Echocardiography: official publication of the American Society of Echocardiography 16, 777–802, doi: 10.1016/s0894-7317(03)00335-3 (2003). 12835667

[b30] Said S. M., Dearani J. A., Ommen S. R. & Schaff H. V. Surgical treatment of hypertrophic cardiomyopathy. Expert review of cardiovascular therapy 11, 617–627, doi: 10.1586/erc.13.46 (2013). 23621143

