# Supplementary material for: Outcomes of an extended Morrow procedure without a concomitant mitral valve procedure for hypertrophic obstructive cardiomyopathy
Source: Sci Rep. 2016 Jun 30;6:29031. doi: 10.1038/srep29031 (PMC4928185; doi:10.1038/srep29031)
Supplement: Supplementary Information [file srep29031-s1.pdf]

Outcomes of an extended Morrow procedure without a concomitant mitral valve procedure for hypertrophic obstructive cardiomyopathy

Yun Liu, MD; Yunhu Song*, MD; Ge Gao, MD, PhD; Jun Ran, MD; Wenjun Su, MD, PhD; Haojie Li, MD, Yajie Tang, MD, Fujian Duan, MD, Hansong Sun, MD

Supplementary Material

Supplementary Table S1, Parameter Used in Grading Mitral Regurgitation Severity.

	**Trace**	**Mild**	**Moderate**	**Severe**
**Color flow jet area**	<10% of LA area and confined to MV orifice	10%-20% of LA area	20%-40% of LA area	>40% of LA area

LA, left atrium; MV, mitral valve.

Supplementary Table S2, Characteristics of HOCM Patients with Hypertension.

Parameters	All patients	Preoperative mild MR	Preoperative moderate or severe MR	P value
Age (yrs)	56.0±7.90	56.0±7.88	56.0±8.02	0.981
Males	45.6(26/57)	68.4(13/19)	34.2(13/38)	0.023
Heart rate (beats/min)	65.4±4.72	66.4±4.96	65.0±4.59	0.295
Systolic blood pressure before admission (mmHg)	115.5±11.6	114.8±10.6	115.9±12.2	0.732
Diastolic blood pressure before admission (mmHg)	72.8±10.3	73.2±10.8	72.5±10.2	0.732
Maximum of systolic blood pressure (mmHg)	166.7±19.4	167.9±16.2	166.0±21.0	0.738
Maximum of diastolic blood pressure (mmHg)	101.3±19.4	101.8±14.6	101.0±21.6	0.879
Duration of hypertension (months)[a]	84(48-192)	84(48-240)	78(46.75-144)	0.671
IVS thickness (mm)	23.7±5.29	22.6±5.63	24.2±5.10	0.268
Use of medication before admission				
β-blocker	100(57/57)	100(19/19)	100(38/38)	
Diltiazem	38.6(22/57)	36.8(7/19)	39.5(15/38)	1.000

ACE inhibitors	33.3(19/57)	52.6(10/9)	23.7(9/38)	0.039
Dihydropyridine derivatives	35.1(20/57)	57.9(11/19)	23.7(9/38)	0.018
Diuretics	22.8(13/57)	15.8(3/19)	26.3(10/38)	0.510

ACE, angiotensin-converting enzyme.

Values are shown as mean±SD or % (n/N).

[a] The value is shown as median and quartiles. The difference between the two groups were tested by Mann-Whitney U test.
